# POPs vs. Fat: Persistent Organic Pollutant Toxicity Targets and Is Modulated by Adipose Tissue

**DOI:** 10.1289/ehp.121-a61

**Published:** 2013-02-01

**Authors:** Julia R. Barrett

**Affiliations:** Julia R. Barrett, MS, ELS, a Madison, WI–based science writer and editor, has written for *EHP* since 1996. She is a member of the National Association of Science Writers and the Board of Editors in the Life Sciences.

Research over the last decade indicates that adipose (fat) tissue serves as more than an energy depot. The tissue has a dynamic role in maintaining normal carbohydrate and lipid levels as well as in regulating metabolic and other physiologic functions. Persistent organic pollutants (POPs), including certain organochlorine pesticides and numerous industrial chemicals, are highly attracted to lipids and accumulate in adipose tissue. A new review examines how adipose tissue both modulates and serves as a target of POP toxicity and highlights knowledge gaps [*EHP* 121(2):162–169, La Merrill et al.].

Adipose tissue contains diverse cell types, including adipocytes (fat cells), preadipocytes (immature fat cells), and immune cells. The cells not only respond to various metabolic signals, such as insulin from the pancreas, but also direct the activities of other cell types within the tissue and throughout the body. Adipocytes alone have multiple roles, including lipid storage, production of hormones that regulate appetite and metabolic functions, and secretion of molecules involved in inflammation, which triggers other metabolic and immune system cascades.

Adipose tissue readily accumulates POPs, environmental contaminants associated with disruption of the endocrine, reproductive, and immune systems, impaired neurobehavioral development, and cancer. In the short term, adipose tissue sequesters POPs, limiting the exposure of other tissues. However, storage capacity and duration are not uniform and may vary by adipose tissue subtype. These factors complicate the task of predicting bodywide distribution of POPs, exposure of other tissues, and the compounds’ eventual metabolism and excretion. Over the long term, adipose tissue could serve as an internal source for chronic POP exposure, particularly with weight loss.

**Figure 1:**
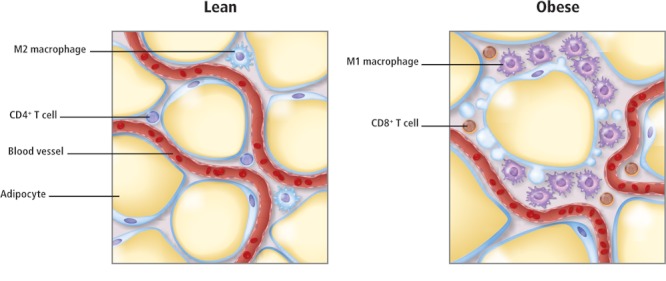
Adipose tissue in lean people is characterized by M2 macrophages and CD4^+^ T cells. In obese people, the tissue accumulates M1 macrophages and CD8+ T cells, signs of inflammation. POPs may also contribute to inflammation in adipose tissue. © 2012 janewhitney.com

Adipose tissue itself may experience toxic effects, especially if exposure occurs within critical windows of susceptibility, such as during prenatal, early postnatal, or pubertal development. Developmental exposure could redirect gene expression, with effects that may not become apparent until later in life. This mechanism, among other possibilities, could explain how several POPs may act as obesogens, compounds that increase the risk of obesity, itself a risk factor for diabetes, liver and cardiovascular diseases, and cancer. Furthermore, recent studies show that POPs provoke an inflammatory state in adipose tissue, a condition associated with the metabolic side effects of obesity. POPs also appear to have a role in lipotoxicity, the accumulation of lipids in nonadipose tissues, leading to metabolic dysfunction characteristic of cardiovascular disease and heart disease.

Ample research points to adipose tissue being a central factor in POP toxicity, but significant knowledge gaps remain with regard to obesogens’ mechanisms of action, POP distribution and dynamics in the body, and the molecular pathways disrupted by or involved in POP toxicity. Human studies, especially prospective longitudinal investigations, are also needed to validate experimental findings.

